# Assessment of Statin Treatment for Pulmonary Alveolar Proteinosis without Hypercholesterolemia: A 12-Month Prospective, Longitudinal, and Observational Study

**DOI:** 10.1155/2022/1589660

**Published:** 2022-10-25

**Authors:** Shenyun Shi, Xianhua Gui, Jingjing Ding, Shangwen Yang, Xiaoyan Xin, Kaifeng Xu, Yonglong Xiao

**Affiliations:** ^1^Department of Respiratory and Critical Care Medicine, Nanjing Drum Tower Hospital, The Affiliated Hospital of Nanjing University Medical School, No. 321 Zhongshan Road, Nanjing, 210008 Jiangsu, China; ^2^Department of Radiology, Nanjing Drum Tower Hospital, The Affiliated Hospital of Nanjing University Medical School, Nanjing, 210008 Jiangsu, China; ^3^Department of Respiratory and Critical Care Medicine, Peking Union Medical College Hospital, Beijing, China

## Abstract

**Background:**

Pulmonary alveolar proteinosis (PAP) is a rare disorder which is characterized by the accumulation of excessive surfactant lipids and proteins in alveolar macrophages and alveoli. Oral statin therapy has been reported to be a novel therapy for PAP with hypercholesterolemia. We aimed to evaluate the safety and efficacy of oral statin therapy for PAP without hypercholesterolemia.

**Methods:**

In a prospective real-world observational study, 47 PAP patients without hypercholesterolemia were screened. Oral statin was initiated as therapy for these PAP patients with 12 months of follow-up.

**Results:**

Forty PAP patients completed the study. 26 (65%) of 40 PAP patients responded to statin therapy according to the study criteria. Partial pressure of arterial oxygen (PaO2) and percentage of diffusion capacity predicted (DLCO%) significantly increased while disease severity score (DSS) and radiographic abnormalities decreased after 12 months of statin therapy (all *p* < 0.05). The factors associated with response were higher levels of granulocyte-macrophage colony-stimulating factor (GM-CSF) antibody and baseline total cholesterol/high-density lipoprotein cholesterol (TC/HDL) (*p* = 0.015 and *p* = 0.035, respectively). The area under the receiver operating characteristic curve (AUROC) of dose of atorvastatin for predicting the response to statin therapy for PAP was 0.859 (95% CI: 0.738-0.979, *p* < 0.001). The cutoff dose of atorvastatin was 67.5 mg daily with their corresponding specificity (64.3%) and sensitivity (96.2%). No severe side effects were observed during the study.

**Conclusions:**

In PAP patients without hypercholesterolemia, statin therapy resulted in improvements in arterial blood gas (ABG) measurement, pulmonary function, and radiographic assessment.

## 1. Introduction

Pulmonary alveolar proteinosis (PAP) is a rare and unique interstitial lung disease characterized by excessive surfactant accumulation within the pulmonary alveoli leading to a variable impairment of pulmonary gas transfer [1]. PAP can be classified into three apparently distinct forms, including primary (autoimmune and hereditary), secondary, and congenital in accordance with the underlying pathogenetic mechanism [2]. Currently, whole lung lavage (WLL) therapy is the standard therapy for PAP [3]. Moreover, it has been reported that inhaled granulocyte-macrophage colony-stimulating factor (GM-CSF) can be an effective therapy for autoimmune PAP in a double-blind, placebo-controlled, and three-group trial [4].

Nowadays, cholesterol was identified to be the most abundant material accumulating within the alveolar macrophages in PAP patients. Oral statin therapy has been considered as a promising new treatment for PAP [5]. Two cases of autoimmune PAP with hypercholesterolemia were reported to significantly improve after statin therapy. However, whether PAP patients without hypercholesterolemia will benefit from statin treatment in a PAP population is still unclear. In this study, we prospectively evaluated the safety and efficacy of oral statin therapy for PAP without hypercholesterolemia.

## 2. Methods

### 2.1. Participants

The study was conducted from March 1, 2019 to December 12, 2021. Patients aged at least 18 years old and diagnosed as PAP were enrolled at the Affiliated Hospital of Nanjing University Medical School. Patients were ineligible if they were pregnant or breast-feeding, had hypercholesterolemia, had chronic lung diseases or any other serious medical conditions, or had been treated with WLL or GM-CSF. The diagnosis of PAP was initiated by chest high-resolution computed tomography (HRCT) scan and confirmed by staining of bronchoalveolar lavage fluid (BALF) or lung biopsy. The study was conducted according to the Declaration of Helsinki and approved by the Ethics Committee of Nanjing Drum Tower Hospital, the Affiliated Hospital of Nanjing University Medical School. The approval number of the ethics committee is 2019-106-01. All subjects gave informed written consent to participate.

### 2.2. Baseline Evaluation

Demographic information, symptoms, and a full blood examination including plasma lipids, serum lactate dehydrogenase (LDH), carcinoembryonic antigen (CEA), and cytokeratin 21-1(CYFRA 21-1) of each enrolled PAP patient had been evaluated. GM-CSF antibody was assayed by the method established by Uchida et al. [6, 7]. The cutoff point was 2.39 g/mL, with measurements in excess of this value resulting to a positive report [8]. Before statin therapy, arterial blood gas (ABG) measurement and pulmonary function tests including forced expiratory volume in one second (FEV1), forced vital capacity (FVC), and carbon monoxide diffusion capacity (DLCO) were performed. Each patient was assigned a PAP disease severity score (DSS) based on the criteria defined by Inoue et al. [9]. The grades ranged from grade 1 to grade 5: grade 1, PaO2 ≥ 70 mmHg without respiratory symptoms; grade 2, PaO2 ≥ 70 mmHg with respiratory symptoms; grade 3, 70 mmHg > PaO2 ≥ 60 mmHg; grade 4, 60 mmHg > PaO2 ≥ 50 mmHg; and grade 5, PaO2 < 50 mmHg. Moreover, quantitative analysis of the HRCT scans including opacification distribution percent and opacification Hounsfield unit (HU) value of percent were evaluated by an automated deep learning artificial intelligence (AI) tool.

### 2.3. Statin Administration

Regardless of the specific form of PAP, oral atorvastatin was initiated as therapy for these enrolled PAP patients. At the beginning of statin initiation, these PAP patients received oral administration of atorvastatin, 20 mg daily. Patients were followed up by visits at 1, 3, 6, 9, and 12 months. Later, the dose of atorvastatin was increased by 10 mg daily to 15 mg daily at each follow-up if patient showed no radiographic improvement including the percentage of lung ground-glass opacification of the whole lung and 5 lobes and the percentage of different densities of ground glass. All patients provided written informed consent for statin therapy.

### 2.4. Monitoring during Treatment

Safety of statin therapy was assessed with symptoms and serum liver enzyme test. Patients were followed up by visits at 1, 3, 6, 9, and 12 months. Symptoms, ABG values, serological measurement including plasma lipids, LDH, CEA, and CYFRA 21-1 and HRCT scanning were obtained at each visit during the study. Pulmonary function tests were just performed at baseline and at 12-month follow-up.

### 2.5. Response Criteria

A complete response was defined as a normalization of HRCT scan, DLCO (within the normal range, the range based on the gender, age, and weight of each patient) and alveolar arterial oxygen gradient (A-aDO2). A partial response was defined as a 50% improvement in one or more of the parameters (total opacification percentage of whole lung in HRCT, DLCO, and A-aDO2) during the study [10].

### 2.6. Statistical Analysis

Continuous variables were expressed as mean ± standard deviation (SD). Differences between the two groups were analyzed by the *t*-test. Categorical variables were expressed as percentages and compared by the Chi-squared test. Changes in PaO2, A-aDO2, and quantitative analysis of HRCT were performed with GraphPad Prism version 7 (GraphPad Software Inc., La Jolla, CA, USA). The relationship between baseline lipid ratio and changes in PaO2, A-aDO2, DLCO%pred, and total opacification of whole lung was assessed by the Pearson correlation analysis. Receiver operating characteristic (ROC) analyses were performed to calculate area under the ROC curve (AUC) of dose of atorvastatin for predicting the response to statin therapy for PAP. A cutoff value that ensured an optimum combination of sensitivity and specificity was calculated. Data were analyzed using SPSS 26.0 statistical software. *p* < 0.05 (two-sided) was considered to indicate statistical significance.

## 3. Results

### 3.1. Baseline Characteristics of Study Participants

**Figure 1:**
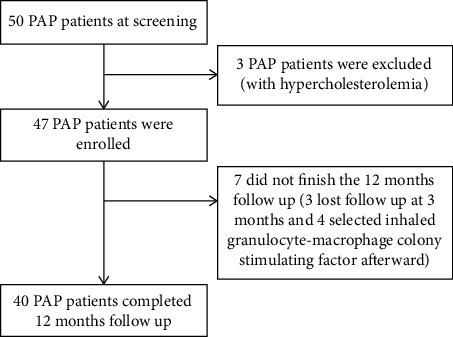
Flow diagram of the study population.

Fifty PAP patients were screened, and 47 patients were enrolled; three of whom were found to be ineligible, diagnosed as PAP with hypercholesterolemia. After 12 months of follow-up, 40 patients (85.1%) completed the study. The period of recruitment and follow-up was from March 1, 2019 to December 12, 2021 after the last enrolled patient completed his 12-month follow-up.  Figure 1 presented the flow diagram of the study cohort.

Of the 40 PAP patients, 33 (82.5%) patients presented with progressive dyspnea, 26 (65%) with cough, and 1(2.5%) with chest pain. Five out of 40 patients were diagnosed by regular health check-up without any symptoms. High-resolution computed tomography (HRCT) of the chest of the 40 patients revealed ground glass opacification and septal thickening. According to the underlying pathogenetic mechanism, 38 patients were classified to autoimmune PAP; one patient was classified to secondary PAP and one patient diagnosed as unclassified PAP.

Clinical and biochemical characteristics of the 40 PAP patients were listed in Table 1. The mean age at diagnosis was 47.70 ± 12.67 years. Predominantly male patients had PAP (55.00%). 14 out of 40 patients were smokers before diagnosis. Mean level of anti-GM-CSF antibody was 27.18 ± 27.56 *μ* g/mL. The mean duration of symptoms was 14.69 ± 21.63 months in our patients. Mean serum TC, CYFRA21-1, and CEA levels were 4.20 ± 0.86 mmol/L, 9.31 ± 8.08 ng/mL, and 3.90 ± 4.33 ng/mL, respectively. Mean DLCO%pred was 60.26 ± 20.90%. Quantitative analysis of lung ground-glass opacification and the density of ground-glass at baseline are shown in Table 2.

### 3.2. Efficacy of Statin Treatment for PAP

At the end of the study, 26 (65%) of 40 PAP patients responded to statin therapy according to the study criteria. Among 26 responding patients, 4 patients had complete responses and 22 patients achieved partial responses. For the 22 patients with partial responses, one patient was classified as a responder on the basis of the radiographic, A-aDO2, and DLCO criteria; 4 patients were classified as a responder on the basis of the radiographic and DLCO criteria; 2 patients were classified as a responder on the basis of the radiographic and A-aDO2 criteria; one patient was classified as a responder on the basis of the A-aDO2 and DLCO criteria; and 14 patients showed radiographic improvement in accordance with the radiographic criteria.

**Figure 2:**
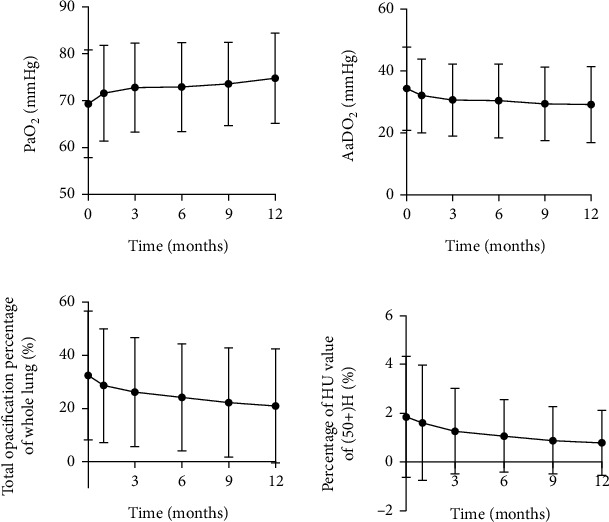
Changes in PaO2, A-aDO2, and quantitative analysis of HRCT from baseline to 1, 3, 6, 9, and 12 months of follow-up.

PaO2 and DLCO%pred significantly increased while disease severity score, total opacification percentage of whole lung, and percentage of HU value of [50+)H levels significantly decreased after 12 months of statin therapy. However, there was no significant difference in A-aDO2 between baseline and 12-month follow-up (Table 3).  Figure 2 showed changes over time in PaO2, A-aDO2, and quantitative analysis of HRCT. No significant difference was observed in A-aDO2 from baseline to 12-month follow-up. Significant difference in PaO2 emerged by the end of the study. [PaO2: 69.31 ± 11.49 vs. 73.60 ± 8.88, *p* = 0.066 (between baseline and 9 months follow-up); 69.31 ± 11.49 vs. 74.78 ± 9.64, *p* = 0.024 (between baseline and 12-month follow-up) .] The changes of total opacification percentage of whole lung and percentage of HU value of [50+)H level showed significant differences by the 9 months follow-up. [Total opacification percentage of whole lung: 32.49 ± 24.15 vs. 24.15 ± 20.07, *p* = 0.097 (between baseline and 6 months follow-up); 32.49 ± 24.15 vs. 22.24 ± 20.37, *p* = 0.044 (between baseline and 9 months follow-up) .] [Percentage of HU value of [50+)H: 1.85 ± 2.48 vs. 1.07 ± 1.49, *p* = 0.091 (between baseline and 6 months follow-up); 1.85 ± 2.48 vs. 0.89 ± 1.36, *p* = 0.035 (between baseline and 9 months follow-up)].

### 3.3. Predictors of Response to Statin Therapy for PAP

The analysis of variables associated with the response to statin therapy is shown in Table 4. The factors associated with response were higher levels of GM-CSF antibody and TC/HDL (*p* = 0.015 and *p* = 0.035, respectively). Responders were observed with higher percentage of HU value of [50+)H in HRCT at baseline. As shown in Table 4, no statistically significant association was identified between efficacy of statin therapy for PAP and baseline level of TC, PaO2, A-aDO2, disease severity score, DLCO%pred, and total opacification percentage of whole lung (all *p* > 0.05).

**Figure 3:**
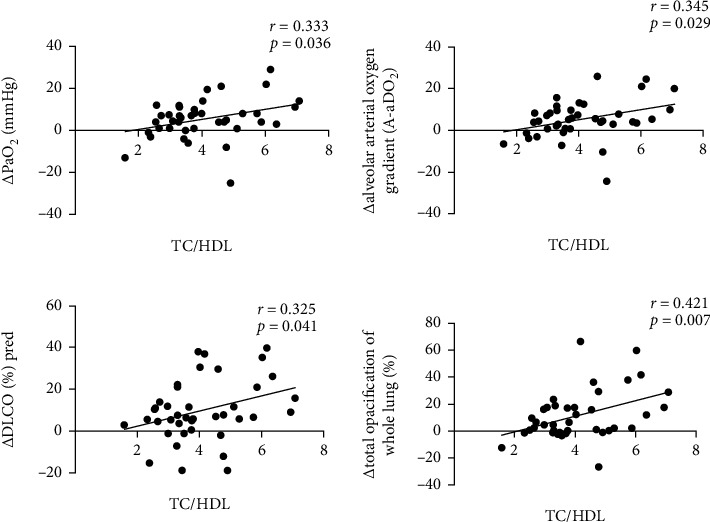
Correlation between baseline TC/HDL level and *Δ*PaO2, *Δ*A-aDO2, *Δ*DLCO%pred, and *Δ*total opacification of whole lung(%) in 40 PAP patients after 12 months of statin treatment.

We further defined the change of PaO2, A-aDO2, DLCO%pred, and total opacification of whole lung (%) after 12 months of statin treatment and at baseline as *Δ*PaO2, *Δ*A-aDO2, *Δ*DLCO%pred, and *Δ*total opacification of whole lung (%). Pearson correlation analysis was evaluated for correlation between baseline levels of TC/HDL, GM-CSF antibody and *Δ*PaO2, *Δ*A-aDO2, *Δ*DLCO%pred, and *Δ*total opacification of whole lung (%). As shown in Figure 3, *Δ*PaO2, *Δ*A-aDO2, *Δ*DLCO%pred, and *Δ*total opacification of whole lung (%) were positively correlated with baseline TC/HDL level (r = 0.333, *p* = 0.036; r = 0.345, *p* = 0.029; r = 0.325, *p* = 0.041; *r* = 0.421, *p* = 0.007, respectively). However, no association was observed between the baseline levels of GM-CSF antibody and *Δ*PaO2, *Δ*A-aDO2, *Δ*DLCO%pred, and *Δ*total opacification of whole lung (%) (*p* = 0.282, 0.363, 0.230, 0.336, respectively).

### 3.4. Dose of Statin Therapy for PAP

**Figure 4:**
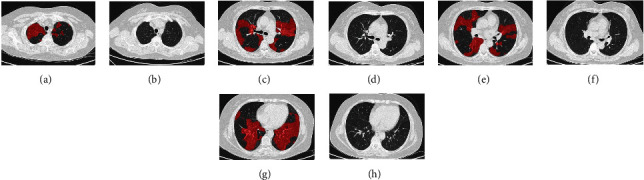
Comparison HRCT scans of one PAP patient with complete response between baseline and 9 months follow-up by AI.

At the end of the study, maximum dose of atorvastatin reached at 75 mg daily. Of 4 PAP patients with complete responses, one patient showed gradual resolution of disease within 9 months of atorvastatin therapy at 20 mg daily (comparison of HRCT scans between baseline and 9 months follow-up by AI shown in Figure 4); one patient showed gradual resolution of disease within 12 months of atorvastatin therapy at 20 mg daily; and the other 2 patients showed gradual resolution of disease within 12 months of atorvastatin therapy at 30 mg daily.

**Figure 5:**
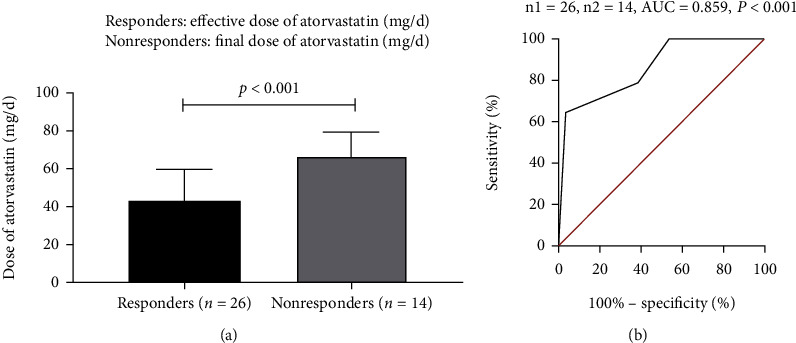
(a) Comparison the dose of atorvastatin between responders and nonresponders. (b) Receiver operating characteristic (ROC) curve of dose of atorvastatin for predicting the response to statin therapy for PAP.

We further defined the effective dose of atorvastatin for responders (*n* = 26) and the final dose of atorvastatin for nonresponders (*n* = 14). Patients who responded to statin therapy received lower dose of atorvastatin than nonresponders (42.88 ± 17.10 vs. 66.43 ± 12.77, *p* < 0.001) (Figure 5(a)). The accuracy of dose of atorvastatin for predicting the response to statin therapy for PAP was then evaluated by the receiver operating characteristics (ROC) analysis. The area under the ROC curve was 0.859 (95% CI: 0.738-0.979, *p* < 0.001) (Figure 5(b)). The cutoff dose of atorvastatin for predicting the response to statin therapy for PAP was 67.5 mg daily with their corresponding specificity (64.3%) and sensitivity (96.2%).

### 3.5. Safety of Statin Therapy for PAP

None of our patients died during the 12-month follow-up. Headache, upper respiratory tract infection, nausea, fatigue, dizziness, and muscle pain were not observed over the treatment of atorvastatin in our 40 PAP patients.

As to liver side effects, 12 of 40 PAP patients had an increase in the alanine aminotransferase (ALT) level (ranging from 40.5 U/L to 83.5 U/L, normal range: 5-40 U/L). There was no significant difference in the occurrence of increase in the ALT level between responders and nonresponders (*p* = 0.347). Taken together, there were no serious side effects in our 40 PAP patients treated with atorvastatin.

## 4. Discussion

In this study, we prospectively evaluated the safety and efficacy of oral statin therapy for PAP without hypercholesterolemia. After 12 months of statin therapy, improvements in oxygenation, DLCO, and radiographic assessments had been observed among patients with PAP. Moreover, no obvious side effects were found by the end of study. The result of this study provides the possibility of statin therapy to be a promising therapeutic approach for PAP without hypercholesterolemia.

WLL which removes accumulated surfactant physically has been the first-line and standard therapy for PAP since the 1960s [3]. It is an invasive procedure almost universally performed under general anaesthesia that can only be performed properly in specialized hospitals. Moreover, WLL is associated with potential complications including infections, fever, convulsions, pneumothorax, pleural effusion, hypoxemia, or even death. It usually provides temporary symptomatic benefit, and about 70% patients need another WLL within 3 years due to recurrence [11]. Given the invasiveness and short-term effectiveness of WLL, new treatments such as subcutaneous or inhaled GM-CSF supplementation, plasmapheresis, or rituximab infusions have been proposed in recent years [12]. A recent double-blind and three-group trial involving 138 patients with autoimmune PAP showed that daily administration of inhaled recombinant GM-CSF led to greater improvements in pulmonary gas transfer and functional health status [4]. However, in a study by Tazawa et al., it was reported that there was a modest salutary effect on the A-aDO2 and CT density quantitative measurement but no clinical benefits of inhaled recombinant GM-CSF in autoimmune PAP patients [13].

A recent study has identified cholesterol as a possible target for a new therapeutic approach in PAP [5]. In our center, one case of PAP without hypercholesterolemia experienced improvements in dyspnea, radiographic abnormalities, and pulmonary function after 18 months of oral statin treatment which already responded poorly to WLL and inhaled GM-CSF supplementation [14]. We observed similarly favorable results in the present study, with a significant difference between baseline and 12-month follow-up in the PaO2, DLCO%, DSS, and radiographic abnormalities. PAP is a syndrome of altered surfactant homeostasis, characterized by accumulation of surfactant lipids and proteins in the airspaces, leading to dyspnea, fatigue, and exercise intolerance. Surfactant homeostasis is maintained by the recycling and catabolism of alveolar type II epithelial cells and the phagocytosis of alveolar macrophages [15]. Statins, as 3-hydroxy-3-methylglutaryl-CoA (HMG-CoA) reductase inhibitors, reduce endoplasmic reticulum (ER) cholesterol and then reducing the cholesterol accumulating within the alveolar macrophages [16]. Ex vivo statin treatment was able to reduce cholesterol accumulation by 40% in alveolar macrophages, which demonstrated that statin had a direct effect on alveolar macrophages [5].

Moreover, the association between higher level of circulating anti-GM-CSF antibodies, baseline total TC/HDL, and therapeutic response was also observed in this study. In a study by Seymour et al., the level of baseline anti-GM-CSF antibody did not differ according to the response to GM-CSF treatment (responders: median 241 *μ*g/mL (range 85-410); nonresponders: median 230 *μ*g/mL (range 51-618), *p* = 0.9, 10]. However, Bonfield et al. have reported that BALF anti-GM-CSF antibody is lower in the responder (*n* = 5) than in the nonresponder (*n* = 6) in terms of GM-CSF therapy [17]. Therefore, correlations between concentration of baseline circulating anti-GM-CSF antibodies and response to statin therapy should be investigated in larger PAP population samples. In the present study, the level of baseline total TC/HDL was higher in the responders than nonresponders. Nevertheless, the levels of *Δ*PaO2, *Δ*A-aDO2, *Δ*DLCO%pred, and *Δ*total opacification of whole lung (%) were positively correlated with baseline TC/HDL level over the statin therapy. It has been indicated that free cholesterol and cholesterol esters were largely increased in PAP patients by lipid compositional analysis [18]. As to the role of lipid ratios in PAP, serum TC/HDL ratios of PAP patients were significantly higher compared to healthy subjects [19]. In addition, the level of TC/HDL-C has been reported to be positively associated with the occurrence and severity of PAP [20]. In the future, we could monitor effectiveness of statin therapy for PAP by detecting the levels of serum TC/HDL ratios.

In terms of safety, statin-induced adverse effects commonly contain liver impairing, bleeding, irregular menstrual cycles, and so on [21]. Less than half of PAP patients had an increase in the level of ALT, and the level of ALT decreased gradually to normal after medical intervention. Thus, statin therapy is a safe and convenient choice for PAP patients.

Our study has several limitations. First of all, this is a prospective, longitudinal, and observational study, lacking the comparison of placebo-controlled. Secondly, our sample size was small. However, considering the low frequency of PAP in the general population, one can consider the sample size in our study as acceptable to assess responsiveness of statin therapy. Finally, data of this study was prospectively collected from a single centre.

## 5. Conclusion

In summary, improvements in oxygenation, DLCO, and radiographic assessments had been observed among patients with PAP regardless of the level of blood cholesterol after 12 months of oral statin treatment. Our study offers the possibility that oral statin therapy might be a promising therapeutic option for PAP.

## Figures and Tables

**Table 1:** The clinical and biochemical properties of 40 PAP patients.

Parameter	PAP (*n* = 40)
Age (year)	47.70 ± 12.67
Gender (female, no. (%))	18 (45.00)
Duration of symptoms (months)	14.69 ± 21.63
Smoking history (current smoking or previous smoking)(Y,%)	14 (35.00)
GM-CSF antibody (*μ* g/mL)	27.18 ± 27.56
TC (mmol/L)	4.20 ± 0.86
TG (mmol/L)	1.75 ± 1.19
HDL (mmol/L)	1.15 ± 0.43
LDL (mmol/L)	2.50 ± 0.75
TC/HDL	4.07 ± 1.34
LDH (U/L)	268.23 ± 90.51
CEA (ng/mL)	3.90 ± 4.33
CYF21-1 (ng/mL)	9.31 ± 8.08
PaO2 (mmHg)	69.31 ± 11.49
Alveolar arterial oxygen gradient (A-aDO2) (mmHg)	34.29 ± 13.48
Disease severity score	2.63 ± 1.01
FVC%pred	79.01 ± 17.78
FEV1%pred	80.19 ± 18.39
DLCO%pred	60.26 ± 20.90

GM-CSF antibody: granulocyte-macrophage colony-stimulating factor antibody; TC: total cholesterol; TG: triglyceride; HDL: high-density lipoprotein cholesterol; LDL: low-density lipoprotein cholesterol; LDH: lactate dehydrogenase; CEA: carcinoembryonic antigen; CYFRA21-1: cytokeratin 21-1; PaO2: partial pressure of arterial oxygen; FVC: forced vital capacity; FEV1: forced expiratory volume in one second; DLCO: carbon monoxide diffusion capacity.

**Table 2:** Quantitative analysis of lung ground-glass opacification and the density of ground-glass at baseline.

Quantitative analysis of HRCT	PAP (*n* = 40)
Total opacification percentage of whole lung (%)	32.49 ± 24.15
Opacification percentage of right upper lobe (%)	35.69 ± 27.76
Opacification percentage of right middle lobe (%)	31.26 ± 24.55
Opacification percentage of right lower lobe (%)	34.29 ± 28.71
Opacification percentage of left upper lobe (%)	31.50 ± 23.51
Opacification percentage of left lower lobe (%)	29.58 ± 26.78
Percentage of HU value of (-∞,-750)H (%)	5.54 ± 5.34
Percentage of HU value of [-750,-300)H (%)	17.83 ± 14.28
Percentage of HU value of [-300, 49)H (%)	7.19 ± 7.20
Percentage of HU value of [50+)H (%)	1.85 ± 2.48

HRCT: high-resolution computed tomography; PAP: pulmonary alveolar proteinosis; HU: Hounsfield unit.

**Table 3:** Comparison of clinical parameters and radiographic assessment before and after statin treatment in 40 PAP patients.

Parameter	At baseline	12-month follow-up	*p* value
TC (mmol/L)	4.20 ± 0.86	3.35 ± 0.75	<0.001
TG (mmol/L)	1.75 ± 1.19	1.33 ± 0.80	0.066
HDL (mmol/L)	1.15 ± 0.43	1.29 ± 0.35	0.092
LDL (mmol/L)	2.50 ± 0.75	1.60 ± 0.64	<0.001
TC/HDL	4.07 ± 1.34	2.68 ± 0.59	<0.001
LDH (U/L)	268.23 ± 90.51	230.28 ± 60.02	0.030
CEA (ng/mL)	3.90 ± 4.33	3.31 ± 2.49	0.459
CYF21-1 (ng/mL)	9.31 ± 8.08	5.87 ± 4.31	0.021
PaO2 (mmHg)	69.31 ± 11.49	74.78 ± 9.64	0.024
Alveolar arterial oxygen gradient (A-aDO2) (mmHg)	34.29 ± 13.48	29.08 ± 12.19	0.074
Disease severity score	2.63 ± 1.01	2.13 ± 0.79	0.016
FVC%pred	79.01 ± 17.78	83.65 ± 17.55	0.244
FEV1%pred	80.19 ± 18.39	83.28 ± 18.02	0.450
DLCO%pred	60.26 ± 20.90	70.15 ± 21.04	0.038
Total opacification percentage of whole lung (%)	32.49 ± 24.15	20.96 ± 21.34	0.026
Opacification percentage of right upper lobe (%)	35.69 ± 27.76	26.51 ± 25.17	0.125
Opacification percentage of right middle lobe (%)	31.26 ± 24.55	19.20 ± 20.78	0.020
Opacification percentage of right lower lobe (%)	34.29 ± 28.71	23.56 ± 27.01	0.089
Opacification percentage of left upper lobe (%)	31.50 ± 23.51	22.11 ± 20.96	0.063
Opacification percentage of left lower lobe (%)	29.58 ± 26.78	20.39 ± 23.76	0.109
Percentage of HU value of (-∞,-750)H (%)	5.54 ± 5.34	5.14 ± 5.26	0.741
Percentage of HU value of [-750,-300)H (%)	17.83 ± 14.28	13.04 ± 13.99	0.133
Percentage of HU value of [-300, 49)H (%)	7.19 ± 7.20	3.47 ± 4.94	0.009
Percentage of HU value of [50+)H (%)	1.85 ± 2.48	0.79 ± 1.34	0.021

TC: total cholesterol; TG: triglyceride; HDL: high-density lipoprotein cholesterol; LDL: low-density lipoprotein cholesterol; LDH: lactate dehydrogenase; CEA: carcinoembryonic antigen; CYFRA21-1: cytokeratin 21-1; PaO2: partial pressure of arterial oxygen; FVC: forced vital capacity; FEV1: forced expiratory volume in one second; DLCO: carbon monoxide diffusion capacity; HU: Hounsfield unit.

**Table 4:** Variables associated with therapeutic response to statin therapy in 40 PAP patients.

Feature	Responders (*n* = 26)	Nonresponders (*n* = 14)	*p* value
Age (year)	49.58 ± 11.19	44.21 ± 14.85	0.206
Gender (female)	9 (34.62)	9 (64.29)	0.072
Smoking history (Y)	14 (53.85)	0 (0.00)	0.002
GM-CSF antibody (*μ* g/mL)	33.34 ± 31.86	15.75 ± 10.48	0.015
TC (mmol/L)	4.19 ± 0.81	4.21 ± 0.98	0.952
HDL (mmol/L)	1.07 ± 0.43	1.28 ± 0.40	0.138
LDL (mmol/L)	2.53 ± 0.74	2.44 ± 0.78	0.707
TC/HDL	4.39 ± 1.41	3.47 ± 1.00	0.035
PaO2 (mmHg)	68.33 ± 11.02	71.14 ± 12.53	0.467
Alveolar arterial oxygen gradient (A-aDO2) (mmHg)	35.91 ± 14.23	31.28 ± 11.88	0.307
Disease severity score	2.69 ± 0.97	2.50 ± 1.09	0.570
LDH (U/L)	281.62 ± 97.28	243.36 ± 73.16	0.206
CEA (ng/mL)	3.95 ± 4.52	3.82 ± 4.12	0.931
CYF21-1 (ng/mL)	9.89 ± 8.78	8.22 ± 6.76	0.539
DLCO%pred	60.04 ± 22.99	60.67 ± 17.15	0.929
Total opacification percentage of whole lung (%)	35.45 ± 23.68	26.99 ± 24.92	0.296
Percentage of HU value of [-300, 49)H (%)	8.26 ± 7.55	5.21 ± 6.26	0.205
Percentage of HU value of [50+)H (%)	2.40 ± 2.82	0.83 ± 1.20	0.018

GM-CSF antibody: granulocyte-macrophage colony-stimulating factor antibody; TC: total cholesterol; HDL: high-density lipoprotein cholesterol; LDL: low-density lipoprotein cholesterol; LDH: lactate dehydrogenase; CEA: carcinoembryonic antigen; CYFRA21-1: cytokeratin 21-1; PaO2: partial pressure of arterial oxygen; DLCO: carbon monoxide diffusion capacity; HU: Hounsfield unit.

## Data Availability

The data are available upon request.

## Abbreviations

GM-CSF antibody: Granulocyte-macrophage colony-stimulating factor antibody
TC: Total cholesterol
TG: Triglyceride
HDL: High-density lipoprotein cholesterol
LDL: Low-density lipoprotein cholesterol
LDH: Lactate dehydrogenase
CEA: Carcinoembryonic antigen
CYFRA21-1: Cytokeratin 21-1
PaO2: Partial pressure of arterial oxygen
FVC: Forced vital capacity
FEV1: Forced expiratory volume in one second
DLCO: Carbon monoxide diffusion capacity.
